# Review: Utilization of yeast of *Saccharomyces cerevisiae* origin in artificially raised calves

**DOI:** 10.1186/s40104-017-0165-5

**Published:** 2017-05-01

**Authors:** Gibson M. Alugongo, Jianxin Xiao, Zhaohai Wu, Shengli Li, Yajing Wang, Zhijun Cao

**Affiliations:** State Key Laboratory of Animal Nutrition, Department of Animal Nutrition and Feed Sciences, China Agricultural University, Beijing, People’s Republic of China

**Keywords:** Active live yeast, Calves, Health, Yeast culture

## Abstract

Yeast of *Saccharomyces cerevisiae* (SCY) origin has over long time been incorporated into domestic animal diets. In calves, several products have offered improved performance and health. Although several types of research have been completed, the mode of action of SCY is not clear in calves. Under this review, we have highlighted the works available in the literature on the use of SCY in calves performance, health, immunity, and the gut environment. Both active live yeast and yeast culture have positive effects on growth, rumen, small intestines, immunity and general health of the calf. Specifically, SCY can improve DMI, growth, feed efficiency and reduce diarrhea in calves. Furthermore, subtle improvements are seen in rumen fermentation (increased butyrate production) and rumen papillae growth. These positive results are, however, more pronounced in calves that are under stress or exposed to significant levels of disease-causing agents. There is a need for further research in areas such as gut morphology, gut microbiology and immunity using latest molecular methods to fully understand how SCY helps the growth and development of calves.

## Background

Improved management and nutrition can promote optimal growth, better feed efficiency and health in young calves [1]. On the other hand, low growth rates can result in underweight at weaning and further post-weaning growth which cannot be compensated through future nutrition [2]. Furthermore, for a sustainable and profitable enterprise the costs of raising replacement heifers and age at first calving should be lower [3, 4]. Extensive research related to calves feeds and feeding is available in literature [5, 6]. Despite the progress made over the past decades, animal nutritionists continue to investigate how different dietary components support the growth and well-being of animals. Research is further moving from general responses to diet such as dry matter intake (DMI), growth and fecal scores to specific areas such as metabolic changes and gut microbiota [7]. For example, *Lactobacilli* and *Bifidobacteria* populations are an important component of a balanced microbiota in the gut [8]; however, stress conditions may result in their decreased number hence setting in the pathogenic micro-organisms. Diarrhea, is a leading cause of death in calves (more than 62% of mortality in dairy calves’ industry; [9]), arises from the abundance of pathogenic micro-organisms such as Enterotoxigenic *Escherichia coli* (ETEC) and *Salmonella* in the small intestine which releases enterotoxins into the gut lumen.

Feed additives are usually used on the farm to improve the performance of young animals [3]. The utilization of antibiotics has offered some of these benefits over many years in calves. However, use of antibiotics in livestock production has become a sensitive issue due to the reported cases of antibiotic resistance to pathogens in humans and calves supplemented with antibiotics in milk [10, 11]. Moreover, there is growing concern held by consumers over their effects on human health [12, 13]. Probiotics and prebiotics have been seen as the best alternative to antibiotic use in young animals [14].

Yeast of *Saccharomyces cerevisiae* (SCY) origin has over long time been incorporated into domestic animal diets [15]. Dairy and beef cattle, pigs, horses, sheep and their young ones, have all shown improved performance when fed SCY as compared to those not fed [16–18]. In mature ruminants [19, 20] both active live yeast (ALY) and yeast culture (YC), have reported improved nutrient utilization, altered rumen fermentation and enhanced production parameters [21–23]. Originally, works on growing animals that were exposed to transport stress showed better growth and health when offered SCY products [24]. Recently, research on the utilization and function of SCY products have been gaining extensive interest in calf nutrition [25, 26].

To the best of our knowledge, no effort has been made to summarize the work that exists in the literature on feeding SCY in calves. Therefore, the purpose of this paper is to highlight the advances that have been made in feeding both ALY and YC and try to point out areas of research that can be exploited to add to the available knowledge and help in the further understanding of how SCY improves calves’ performance and health. Some of the discussions incorporate other animal models, due to the paucity of information on studies in calves and with the view that some of the principals can be applied to calves. This review has utilized fully published research as well as research published in abstract form and available online. Our discussion is further limited to only products that contain ALY or YC but where applicable, references have been made to research on products that are derived from *Saccharomyces cerevisiae*.

## Differences in active live yeast and yeast culture

Yeast of *Saccharomyces cerevisiae* origin has been extensively researched on both in-vitro and in vivo [27] to determine its effect in animal models. There are various ALY and YC products from *S. cerevisiae* yeast available on the market. The products are classified based on the active ingredients and their modes of action [20]. Two broad groups exist in the literature depending on the viability of cells in the product. Active live yeast products are fermentable living yeasts that have been dried and contain at least 15 × 10^9^ live yeast cells per gram while a YC is produced through fermenting cereal grains in a selected liquid with bakers yeast then drying the whole medium culture [20, 27]. The YC contents may include yeast cell wall (β-glucans and mannan-oligosaccharides), cell solubles, vitamins, proteins, peptides, amino acids, nucleotides, lipids, organic acids, esters and alcohols, B vitamins, polyphenols, organic acids and anti-oxidants [28, 29] all of which may have positive effects on performance and health when incorporated into the diet of animals. The composition of each of the above bioactive compounds in SCY have not been characterized [30] and as a result, the effects of SCY are mostly attributed to the yeast wall components. Any efforts that would be made to characterize the quantity of the mentioned components would make it comparatively easy while explaining the molecular or physiological changes that are observed in calves.

The active live yeast is considered to offer mainly probiotic effect while the yeast culture components are regarded as having both probiotic and prebiotic effect. Fuller [31] defined probiotics as live microbial supplements that beneficially affects the health and well-being of the host animal by improving its gastrointestinal balance. Recent research has shown that the two products might not have significant differences in their mode of action on rumen fermentation [27].

## Effects of SCY on calf feed intake, growth, feed efficiency

*Saccharomyces cerevisiae* yeast products have the ability to stimulate starter intake in calves (Table 1). However, the impact of feeding SCY on DMI in calves has not been consistent. Experiments have reported different performances both before and after weaning. Some researchers have reported significantly higher intakes pre-weaning [32, 33] while others did not observe any significant changes [34]. Others observed positive effects of SCY on DMI post-weaning only [35]. Several of the papers retrieved reported no differences in DMI whether in the pre-weaning or post-weaning period [36–39]. Different factors such as the strain of yeast, the nature of the diet or the physiological status of the animal [40], dose and feeding strategy [35, 41] influence DMI. The high DMI reported in Harris et al. [26], Galvao et al. [32], and Lesmeister et al. [35], respectively,  happened during periods of the high incidence of diarrhea and abrupt weaning respectively. Using 512 animals, Magalhães et al. [41] found no significant differences between treatment and control groups in both pre- and post-weaning periods. These authors attributed the results to less starter intake in the first 21d of the life of the calf which is very crucial period. In older transition dairy cows, Zaworski et al. [17] have suggested that YC can improve dietary energy utilization or absorption that may be dependent or independent of DMI.

**Table 1:** Showing effects of *Saccharomyces cerevisiae* Yeast (SCY) products in calves performance

S/No	Study	No. of Calves	Period, d	Parameters								
				ADG^1^			DMI^2^, kg			FE^3^		
				CON	SC1	SC2	CON	SC1	SC2	CON	SC1	SC2
1	Lesmeister, et al. [35]	75	0–35	0.38	0.36	0.42	0.28	0.29	0.32	2.73	2.90	2.71
			35–42	0.74^a^	0.92^b^	0.94^b^	1.33^a^	1.42^ab^	1.54^b^	2.33	1.95	1.67
			0–42	0.44^a^	0.44^ab^	0.51^b^	0.48^a^	0.51^ab^	0.56^b^	2.25	2.25	2.00
2	Galvao et al. [32]	52	5–42	0.30	0.47		0.44^a^	0.68^b^		0.27^a^	0.40^b^	
			43–84	0.91	1.04		2.19^a^	2.58^b^		0.43	0.43	
3	Quigley, et al. [34]	42	1–42	0.18	0.17		0.21	0.24		0.30	0.27	
			43–84	0.48	0.51		1.47	1.52		0.31	0.32	
			1–84	0.33	0.43		0.84	0.88		0.30	0.30	
4	Magalhães, et al. [41]	512	4–28	0.23	0.20		0.15	0.13		1.50	1.22	
			29–70	0.78	0.77		1.43	1.41		0.56	0.57	
			4–70	/	/		0.92	0.90		/	/	
5	Panda et al. [39]	12	1–91	0.34^a^	0.48^b^		512	581		3.12^a^	2.49^b^	
6	Harris, et al. [26]	60	0–35	0.48^a^	0.48^a^	0.58^b^	0.18^a^	0.22^ab^	0.25^b^			
7	Hill, et al. [44]	116	3–63	0.51	0.54		0.86	0.81		0.70	0.66	
8	Zhou^4^ et al. [45]	18	1–63	0.31^a^	0.52^ab^	0.66^b^	1.01	0.95	1.01			
9	Seymour, et al. [38]	42	1–46	0.46	0.43		1.04	0.97		2.48	2.55	
10	Brewer^5^ et al. [25]	40	0–14	5.3	5.5		/	/		/	/	
			15–35	10.9^a^	17.4^b^		/	/		/	/	
			0–35	16.7^a^	23.8^b^		/	/		/	/	
11	Yan et al. [53]	12	1–60	0.52^a^	0.66^b^		0.95^a^	1.08^b^		/	/	
12	Hučko, et al. [37]	30	4–56	0.384	0.381		0.61	0.62		2.63	2.96	
13	Hoseinabadi^6^, et al. [80]	30	13–65	0.67	0.67	0.65	1.22^a^	1.00^b^	1.15^ab^	0.39	0.44	0.41
14	Pinos-Rodriguez, et al. [33]	16	4–60	/	/		0.84^a^	0.91^b^		3.46	3.51	
15	Huuskonen and Pesonen [36]	40	20–75	0.82	0.76		0.69	0.67		3.26	2.21	
16	Kaldmäe, et al. [87]	20	6–35	0.38	0.35		1.25	1.22		3.26	3.51	
			36–65	0.99	1.03		2.29	2.33		2.30	2.25	
			6–65	0.69	0.69		/	/		/	/	

*CON Saccharomyces cerevisiae* Yeast (SCY) in milk or starter, *SC1 Saccharomyces cerevisiae* Yeast (SCY) at level 1 unless stated otherwise, *SC2 Saccharomyces cerevisiae* Yeast (SCY) at level 2 unless stated otherwise

^a,b^Significantly different (*P* < 0.05)

*ADG* ^1^ Average Daily Gain

*DMI* ^2^ Dry Matter Intake

*FE* ^3^ Feed Efficiency (DMI/ADG; DMI does not include milk or milk replacer intake)

Zhou^4^ included CON, Starter + hay and Starter + SCY respectively

Brewer^5^ instead of ADG he reported the total body weight gain

Hoseinabadi^6^ included CON, SCY in starter and SCY in milk, respectively

Average daily gain (ADG) is directly related to DMI. With higher DMI, it is likely that the ADG will also be higher (Table 1). However, similar to the lack of changes in DMI in most researches, ADG was not significantly different except for few experiments that had significantly higher ADG post-weaning [35, 39], which were concomitant with DMI. It is, however, possible to have periods of higher ADG in SCY supplemented calves as was observed in a recent research where calves were infected with *Citrobacter freundii* on 16 d of the study [26]. The calves that were supplemented with SCY showed higher ADG during 15 d – 21 d and also had lower fecal scores during the same period. This implies that calves might have been protected from the adverse effects of diarrhea in that window of the period. In an experiment carried out on Yea-Sacc 1026, in calves averaging 54 kg at the beginning of the experiment, calves that had been supplemented with 0.0625% and 0.125% of the concentrate diet gained only 5 kg more than the unsupplemented group at the end of 84 d experiment [42]. No differences have been observed in feed efficiency for calves fed YC [35, 41] probably due to the lack of significant differences in the DMI and ADG in the experiments that reported on FE. In an experiment with an ALY, Panda et al. [39] showed that cross-bred calves without supplementation had a lower feed to gain ratio compared to supplemented group. In lambs, Haddad and Goussous [43] indicated that YC had an effect on body weight, growth and feed to gain ratio though no differences were observed in DMI.

Occasionally, body weight and body structure measurements have also been reported. Hill et al. [44] did not observe any changes in height and heart girth. However Zhou et al. [45] reported that YC improved the height and withers width in calves. Similarly, we did not observe any changes in calves’ withers height, length, heart girth and hip width [46]. The structural changes reported by Zhou et al. [45] could be linked to increase in energy intake and protein as a result of significant increase in digestibility of nutrients. Some authors have argued that SCY can cause an increase in energy and other nutrients intake [35].

Metabolic responses are valuable in providing information on the nutritional status of the animal. For example, glucose and BHBA are good indicators of energy metabolism in calves. However, SCY does not seem to have an effect on blood parameters of calves except for one experiment that showed that a live yeast product can increase glucose concentration [32, 41].

## Effect of SCY on selected health parameters

A calf in good health will have high growth rates and hence better future performance. Calves are very susceptible to diseases caused by various pathogens and environmental stressors in the first months of their lives since their immune system is still naive. Similarly, the weaning process might be very stressful, leading to increased respiratory problems in calves. Low growth rates due to disease can result in poor weaning weight and further post-weaning growth which cannot be compensated through future nutrition [2]. The farmer needs a veritable security against a potential outbreak of gastrointestinal and respiratory diseases which are common in the first couple of months.

The most reported health effects of SCY have been on either the reduction of diarrhea and improvement of fecal scores (Table 2; [32, 38, 41]). In some studies, no effect on health parameters was reported [35]. The reasons for inconsistency in response to supplementation have only been speculated in most of the trials conducted. In an experiment working with 512 calves, 12.1% and 7.5% of control and YC treatment calves died respectively [41]. However, the risk of death was similar before d 13 but 6-fold more among control group afterward. The authors attributed the observations in first two weeks to the low feed intake during this period as the YC was added in calf starter only. In another experiment, effects of anti-*Salmonella* by *Saccharomyces cerevisiae* fermentation product (SCFP), a YC, were investigated by adding the products to milk replacer and starter grain [25]. The calves were fed two weeks before and three weeks after experimental challenge with *Salmonella enterica* serotype *Typhimurium*. Calves were monitored for clinical signs and parameters associated with Salmonellosis after the challenge. Calves that were supplemented with SCFP showed fewer bouts of diarrhea and fever. In the control group, *Salmonella* shedding could be seen 4 d after shedding in the treatment group had stopped. Galvao et al. [32] observed reduced number of days with diarrhea, but the fecal scores were similar to the treatment and control. To investigate whether there were differences between pre- and post-weaning, supplementing an ALY in the feed resulted in a positive effect on the health of the calves before weaning [38]. The positive results observed with SCY in the trials above could be dependent on the level of pathogenicity, the housing conditions, feeding regimes and environmental factors among others.

**Table 2:** The health parameters affected by yeast products in calves

No.	Parameter	Response to SCY supplementation		
		Increased	Similar	Reduced
1.	Diarrhea		[33, 34, 38]	[25, 32, 35, 37, 41, 47, 63, 80]
2.	Fever		[38, 44]	[36]
3.	Respiratory infections		[34, 38, 44]	
4.	Use of antibiotics			[41, 44]
5.	Use of electrolytes		[50]	
6.	Immune responses			[41]
7.	Mortality			[41]
	Medication costs		[50]	[44]
8.	Others (e.g. bloat, cough)		[41]	

The complex stable microbial flora present in the gut helps the animal to resist infections [31]. Modern trends in conditions used to rear animals can disrupt the natural condition that exists in the gut hence interference with animal performance [31]. In young calves, when the pathogenic (such as coliform and lactic acid bacteria) bacteria proliferate in the small intestines, bacterial diversity is interfered with and subsequently reduction in commensal bacteria may arise [47]. Since the decrease in microbial diversity in the first month has been associated with diarrhea incidences in calves [48] could feeding SCY help improve its microbial diversity? Yeast culture metabolites possess the ability to inhibit pathogenic flora while supporting the commensal bacteria in vitro [49]. This could be a major factor in clearing the gut lumen of pathogenic bacteria and hence supporting the commensal bacteria which are important in the process of carbohydrates fermentation that result in higher volatile fatty acids [25]. In large intestines, YC increased the microbial species richness and stimulated the fibrolytic bacteria (*Ruminococcaceae*) colonization, which increased butyrate subsequently lowered diarrhea incidences [46, 50].

Body temperature is an important parameter in diarrheic calves. Fever is usually observed in calves with the very loose fecal matter. Hill et al. [44] observed lower temperatures in Jersey’s calves with a tendency towards significance for calves that had been supplemented with an AYC while Seymour et al. [38] reported significantly lower temperatures for Holstein calves supplemented with YC. Most of the observations were based on a judgment by the eyes for example assigning of fecal scores which are subjective to the person involved in the recording the scores.

Since diarrhea is closely associated with loss of electrolytes, several experiments have shown that yeast products can lead to decreased use of electrolytes in calves [41, 50]. Although the reduction in the use of electrolytes has been reported based on the quantity used more research should be done to determine how SCY reduce losses of electrolytes and reduce dehydration in calves. The intestinal epithelium is the first protective barrier from exogenous pathogens [51]. Calves might have a compromised intestinal permeability even before being given colostrum which subsequently predisposes the calf to higher diarrhea rates even when they have achieved passive transfer of immunity [52].

Calf diarrhea is also an expensive venture in terms of time and labor costs spent on therapy. The SCY can reduce calf raising costs [41, 53] mainly through lower costs on medications. This is especially important on farms with high antibiotic use as evidenced by reduced days of antibiotic therapy in morbid calves and lambs [50, 54, 55] and labor costs. The product is cheap and can be used throughout the growth period with less addition on calf raising costs [41]. Yan et al. [53] observed that it cost less to raise calves supplemented with a YC (20 g/d) by up to 29.98% in a 60 - d experiment. Another advantage of YC is that it can be used in combination with other additives such as ionophores and antibiotics and still be efficacious [56, 57].

Most of the scientists argue that yeast products might be more efficient when animals have been challenged by disease or stressful environment such as weaning stress or when they have been offered high-concentrate diets [40]. In Quigley et al. [34] experiment they concluded that effects of YC on calves was masked by high incidences of disease in calves. While this might have been true, then it is important to note that the SCY products are also evolving with a better understanding on how they may be offering benefits in animals. The extent of stressors and disease might be one of the determinants of the outcome of a test of SCY in calf health [40]. Lack of statistical differences in fecal scores as indicated by Lesmeister et al. [35] is paradoxical but might be as a result of how diarrhea rate was calculated in their experiment or the causal agent of diarrhea in this calves.

## Effect of SCY on calf immunity

The gut provides the site for nutrients absorption and is also the first line of defense against pathogens and other harmful substances for the animal [17]. It remains as the main anatomical location that SCY might play a significant role in immunomodulation. Several complex polysaccharides found in the yeast cell wall such as β-glucans and mannan-oligosaccharides have been identified as the modulators of immunity [29]. The in vitro experiment by Jensen et al. [49] showed that a YC product could provide anti-oxidant, anti-inflammatory and immuno-modulatory activities. Since then, several experiments have been carried out in calves [26], lambs [58] and piglets [59, 60] to determine the efficacy of these SCY products in vivo. Magalhães et al. [41] tried to test some measures of the innate immune system in vivo and found no influence of feeding YC product in calves. Anti-OVA IgG concentrations and phagocytic activity of non-pathogenic *E. coli* were similar between control and YC treated groups. They, however, observed a slight increase in phagocytic activity of neutrophils when cells were incubated with pathogenic *E. coli*. The authors concluded that oligosaccharides present in YC could have enhanced the neutrophils phagocytic activity. However, the mechanism by which the oligosaccharides, in this case, β-glucans stimulated the immune system were not reported. For immunomodulatory effect, β-glucans have to move from the lumen and interact with the immune cells. It has been suggested that cells of *S. cerevisiae* yeast cannot penetrate the intestinal endothelium barrier while only pure β-glucan have immunomodulatory effects [61]. Therefore, it is likely that the whole yeast cell might not stimulate the immune system, but either β-glucan fragments or other alternative mechanisms are involved.

**Fig. 1:**
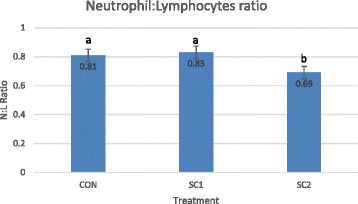
Effects of SCY on neutrophil: lymphocytes ratio in calves. CON: No *Saccharomyces cerevisiae* Yeast (SCY) in milk or starter; SC1: *Saccharomyces cerevisiae* Yeast (SCY) in milk only; SC2: *Saccharomyces cerevisiae* Yeast (SCY) in milk and starter. ^a,b^Significantly different (*P* < 0.05). Adapted from Harris et al. [26]

The yeast cell wall’s β-glucans component are identified as pathogen-associated molecular patterns (PAMPs) by the pattern recognition receptors (PRR) in the lumen [62] since the animal body can neither synthesize them nor are they part of its body. The innate immune system components such as neutrophils, macrophages and natural killer (NK) cells are involved in this response [62]. Recently, Harris, et al. [26] reported a treatment and a time interaction in neutrophil to lymphocytes ratio (NLR) in calves infected with *Citrobacter freundii* and a lower NLR in calves supplemented with a YC (Fig. 1). Similar results have been demonstrated in piglets that had been exposed to weaning and transport stressors [60]. A trial with either healthy or calves infected with an endotoxin and fed with an AYC showed that calves that had been supplemented recovered quickly from the effects of endotoxin compared to the unsupplemented group [63]. When calves are exposed to an endotoxin, they will show clinical signs as a result of changes in hematology such as leukocytes count [64] and in behavioral changes such as those of feed intake, lying or standing time, self-grooming and rumination [65]. Wang et al. [63] results are indicative of the capacity of YC to ameliorate effects of endotoxemia in calves through immunoregulation. Alternatively, the advantage of SCY could come through activation of the adaptive immune responses that require to be activated and proliferate to reach a critical mass that can deal with infectious agents. It also requires to generate effector mechanisms that are most suited to eliminate the infection and this also takes time.

Lack of systemic changes may explain the difficulty in observing the changes in immune parameters [30]. This might be explained by the “dampened” feedback mechanism in the in vivo immune responses that protect the host against excessive inflammatory responses [62]. Moreover, modulation of the immune responses does not lead to excess stimulation or suppression of the immune activity [66]. Immune responses in pre-ruminant calves could also be more dependent on the plane of nutrition. Some researchers have suggested that improved health in calves is based on improved supply of nutrients rather than changes in components of the immune system [67, 68]. In others, neutrophil responses were shown to be higher in calves that received low plane of nutrition compared to the higher plane of nutrition [69, 70]. When taken together, these results suggest that for SCY to be more potent, the animals should be under a certain degree of stress that demands more energy from the animal, which might not have been the case in most of the reported experiments.

Immunological and anti-oxidation systems are mutually complementary mechanisms. In an in vitro study with yeast culture product, Jensen et al. [29] have shown that YC at the lowest level (0.0001 mg/L) can significantly reduce the formation of reactive oxygen species (ROS) in polymorphonuclear (PMN) cells. The YC can be important in animals that experience inflammatory responses in the gut due to pathogenic gut microorganisms [41]. Farmers are advised to check whether the calves have received immunity in the first 24–48 h [71] although this might not be practical on all the farms.

### Future research

There is more exciting information that needs to be unraveled on the effect of SCY in calves’ health. Future studies should focus studying the establishment of intestinal microbiota in young calves supplemented with SCY to help determine the protective capacity of these products as young calves lack a well-developed and stable intestinal microbiota. Since it has been suggested that the yeast components such as the β-glucans, nucleotides or small peptides can directly influence the immune system or indirectly by altering the gut environment [30] and might be more potent in young animals [62] more research need to be done in these areas.

The gut absorptive capacity has been linked to the integrity of the epithelium, while SCY has been shown to improve the crypt depth to villus height ratio [46]. More research using xylose absorption test to determine how SCY reduces the loss of electrolytes and hence fewer electrolyte in calves could be adopted. Xylose absorptive test has been recommended for testing absorptive capacity in the small intestines [72].

Experiments should also consider microbiological analyses of fecal matter to determine the infectious agents available in the environment of the study. It is encouraging, however, to note that the positive effects are present throughout the first three months of life of the calf. More controlled and focused work need to be done to decipher how SCY might affect the general health and immunity of calves. Furthermore, the products should be experimented with animals that have not been exposed to disease causing agents in order determine whether the products can have effect on healthy animals.

## Effects of SCY on gastrointestinal parameters

The stomach of a newborn calf is likened to that of a monogastric animal due to its small and nonfunctional rumen. As the calf ages, the gut anatomy and metabolic functions are expected to change. Calves are fed restricted amounts of liquid feed and starter mixtures containing carbohydrates that are rapidly fermented to butyric and propionate acids. Intake of grain has been shown to support rumen development through production of higher amounts of butyrate. The gut microbiota of a 1–4 weeks old calf is very different from that of advanced age when the rumen is fully developed [47]. However, depending on how early the calf is introduced to a calf starter, it is likely to develop a population of bacteria by 9 wks that is similar in every aspect to that found in a mature animal within the same environment [73]. Moreover, a growing body of evidence shows that microbial colonization in rumen occurs immediately after birth and some rumen bacteria that are essential for mature rumen function are present as early as 1 d after birth [74].

A literature search has shown that SCY mostly affects the rumen and its environment in the older ruminants. In their review, Chaucheyras-Durand et al [40] concluded that SCY carried out its functions primarily by altering the rumen microbial populations. These authors noted that the rumen microbiota could be influenced in three ways: 1. Enhancement of rumen maturity through the favoring microbial establishment, 2. Stabilizing ruminal pH and interactions with lactate-metabolizing bacteria, and 3. The increase in fiber degradation and interactions with plant cell wall degrading micro-organisms.

In vitro, studies have shown that yeast from *Saccharomyces cerevisiae* can increase rumen total bacteria, fungi and protozoa [75] or stimulate fungi [76]. The benefits of SCY were more pronounced in diets that had higher fiber content [28]. In young calves, SCY has also been shown to have an effect on gut microbiota and morphological development, though research in this area is still scarce.

### Ruminal microbiota composition and fermentation patterns

From the available studies, there exist contradictory reports on the effect of SCY on ruminal microbial populations and fermentation patterns. While some researchers have indicated that SCY can positively influence ruminal microbiota in young ruminants [77] others have found none in supplemented calves when compared to control groups [47]. Rumen microbiota in calves responds to dietary modifications, structural and physiological changes in the host animal [73]. The total number of bacteria increased in heifers fed SCY [22]. Ciliate protozoa increased in lambs [55]. These experiments might not reflect the changes that happen in the microbiota of young calves since they used samples from older animals or different species respectively, however, they suggest that SCY might similarly influence the gut microbiota in calves.

Diet composition has an effect on how SCY may affect microbial diversity [22]. In vitro, SCY can stimulate the growth of several bacteria in the rumen, especially lactate utilizing bacteria and those that digest cellulose [28]. In a meta-analysis on feeding an SCY in lactating cows, Robinson and Erasmus [23] proposed that SCY may act by stimulating rumen microbes that increase fermentability of fiber and not by allowing rumen microbes to metabolize more efficiently end-products of ruminal starch fermentation since no impact was noted with increased dietary starch levels. Calf starters are high in grains that have large amounts of starch and lack of reported effects on rumen microbiota might be due to the observations aforementioned. Since the microbial populations in the calves change with age, it would be interesting to investigate the changes that happen with age in rumen microbial flora when calves are fed SCY. Chaucheyras-Durand et al. [40] established that ALY can accelerate microbial diversity in calves which are critical in achieving a functional rumen ecosystem at weaning. Since SCY does not only stimulate bacterial activity in the gut but also change the composition of the bacterial population, current molecular techniques such as high-throughput sequencing can be helpful in identifying those populations influenced by SCY supplementation. In our laboratory, we found out that *Firmicutes*, *Bacteroidetes*, and *Actinobacteria* were predominant throughout the gastrointestinal tract (GIT) of pre-weaned calves, and that bacterial community was highly variable among different GIT sites by using high-throughput sequencing techniques [46]. The inclusion of YC positively affected *Butyrivibrio* richness (fibrolytic bacteria) in rumen liquid in the first 28 d and lowered *Prevotella* on d 28 and d 56 in the supplemented calves which resulted in higher butyrate concentration.

**Fig. 2:**
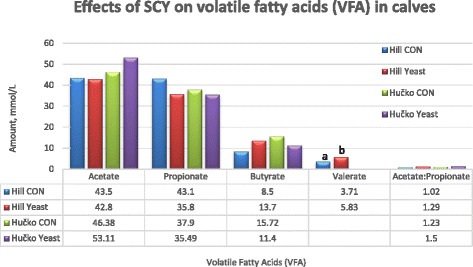
Effects of SCY on volatile fatty acids (VFA) in calves. CON: Not supplemented with *Saccharomyces cerevisiae* Yeast (SCY); Yeast: Supplemented with *Saccharomyces cerevisiae *Yeast (SCY). ^a,b^ Significantly different (*P* < 0.05). Adapted from Hučko et al. [37] and Hill et al. [44]

The effect of SCY on rumen volatile fatty acids (VFA) concentration has not been exhaustively studied in pre-weaning calves. Some researchers have reported decreased total VFA concentration and molar proportion of butyric acid while increasing the molar proportion of acetic acid and the acetate to propionate ratio with live yeast ([37]; Fig. 2). The authors suggested a shift in metabolic activities of ruminal microbiota as a reason for the observed increase in acetate which was attributed to the cellulolytic microbiota. On the other hand, Hill et al. [44] reported no effect in the most important SCFA that is butyrate and propionate in calves that had been supplemented via milk feeding with live yeast at 4 g/d. However, they reported significant differences in valerate concentration, being higher in yeast supplemented calves (3.71 for CON vs. 5.83 for AYC). Most of the available research has been done among older calves, lambs or cows. Mutsvangwa et al. [78] fed a live yeast culture to 3 months old bulls and they reported an increase in concentrations of acetate and total VFA as a result of YC stimulating rumen fermentation. These bulls received a high concentrate diet of barley grain and soya bean meal and barley straw. Although there may be some similarities in these experiments, diets that were given to older animals may contain a high amount of fiber [28] thus contributing to the positive effects observed. Tripathi et al. [55] reported decreased the concentration of VFA’s in lambs attributing the drop to higher rumen fermentation rate and viable bacterial population after SCY supplementation. The same authors did not confirm the ability of live yeast to scavenge oxygen with reported lack of differences in the establishment of fibrolytic bacteria. The microbiota cellular activity measured by enzymatic profile has shown that yeast culture has an effect on some of the short-chain fatty acids (SCFAs) in young ruminant animals [55].

A stable rumen pH in a pre-ruminant calf helps provide a suitable environment for a normal functioning of the rumen bacterial and protozoan population [79]. Ruminal pH can be stabilized through reduced lactate production. Live yeast has been shown to have positive effects on the ruminal parameters of the ruminants [19]. These authors reported that live yeast supplementation led to an increase in ruminal pH and VFAs concentrations while decreasing lactic acid concentration. In pre-ruminant calves, pH seems not to be influenced by YC [46, 80].

### Post-ruminal digestion

There is very scarce information about the effect of SCY on hindgut digestion in ruminants. Research done in horses fed high starch diet reported similar results to those in ruminants when the horses were fed a live yeast [18]. The lack of information in this section of the animal could be related to the assumption that yeast culture functions mainly in the rumen. However, Durand-Chaucheyras et al. [81] have shown that yeast cells remain alive during the transition in the digestive tract leading to the hypothesis that effects of live yeast may extend beyond the rumen. Recently nutritionists have been trying to offer SCY that bypasses the rumen into the hindgut. Milk drank by the calves, bypasses the rumen and enters directly into the abomasum. Hill et al. [44] supplemented live yeast to the calves in milk at the rate of 4 g/d.

### Ruminal and intestinal structural development

Understanding both upper and lower gut development is crucial for the improvement of growth and health of the calf. This development depends on the establishment of ruminal flora, initiation of feed consumption, absorption processes and absorption mechanisms [82]. The VFAs especially butyrate are important in triggering rumen papillae growth [83] which provide the surface area for absorption of nutrients. Recently researchers have demonstrated the importance of introducing starter in calves at an early age. Malmuthuge et al. [84] investigated the effect of feeding milk replacer alone or milk replacer with calf starter combined on microbial gut diversity. They were able to show that calf starter in addition to milk replacer resulted in changes in microbial diversity and expression of gene encoding gut barrier function. Earlier, Tajima et al. [85] showed that diet can have significant effect on rumen microbial composition using real-time PCR method. Diet manipulation through feed additives can further enhance the changes in the gut parameters [86]. There is varying information on the effect of SCY on the structural and functional development of the rumen. Supplementation of the YC at 2% might slightly improve rumen development [35]. These authors observed an increase in papillae length and papillae width at the time of weaning off at 19 and 21% respectively, though not significantly different. They concluded that the number of calves used and the age at which the observations were made could have contributed to the lack of significant differences. Kaldmäe et al. [87] observed no differences in calves’ papillae length and width, rumen wall thickness and a number of papillae at either 1 month or 2 months of age. Feeding a YC to calves has been shown to improve rumen papillae maturation [25] in sick calves. However, Magalhães et al. [41] implied that YC has no effect rumen development and function citing similar grain intake and plasma BHBA levels. On the other hand, effects of yeast on rumen are only likely to be observed if the product is fed through a starter and not milk that bypasses the rumen into the abomasum [44]. Work concluded in our research group showed an improvement in rumen papillae and width [46].

Gut development in the first few weeks of calf’s life is dynamic and can digest and absorb nutrients [88]. Effect of SCY on the ileal mucosal development in calves has not been reported. However, research in poultry showed that whole yeast cells can influence the villus and villus to crypt depth ratio (VCR) ratio when supplemented in the first 21 d.

It is a probability that SCY would function more in the hindgut by reducing the infectious agents. Consequently, if it has to be utilized then bypass method, that is, liquid supply would be more efficient. Similarly, calves younger than 3 weeks of age might not consume more yeast through the starter. The changes observed in morphological changes might be observed mainly after the calf starts consuming a significant amount of the starter [35, 46]. These will be confirmed when rumen development is observed in younger calves such as at 4 weeks of age.

#### Future research

Continued research on the effects of nutrients on gut development is paramount to understanding specific calf management strategies that can be put in place to enhance calf gut health and hence performance [89]. Microbial colonization is fundamental for the growth, development and function of the rumen [73] and is dependent on the diet [82]. Some probiotics have been shown to offer protective functions on cell junctions and mucosal barrier damaged by enterotoxigenic *E. coli* and *Salmonella typhimurium* infection [90] which we have seen can be ameliorated by SCY [25]. Future research might seek to elucidate how SCY might affect the tight junctions in calves. Due to the complexity of the calves’ microbiota focusing on few or individual bacterial species of importance in calves could expedite our understanding of how SCY contributes to calf performance [91]. Similarly, mRNA gene expression could also be used as a tool for changes in crypt depth have been associated with enhanced apoptotic rate [92].

Research should also focus on the influence of SCY on both digesta- and mucosa-associated microbial population and metabolites produced in the GIT. Moreover, we suggest that transcriptomics should be used to elucidate the functional microbial population that might be affected by live yeast and yeast culture supplementation. Making observations over a short period may be helpful in determining at what stage in the life of a calf is YC more effective on structural parameters.

## Suggested mode of action

Both in-vitro and in vivo experiments have been carried out to define the mechanisms of SCY action in animals [27]. Although the mode of action of the SCY has not been fully elucidated [32] its positive results in young animals are encouraging more research in using calves as models to study its effects. Most of the results have been limited to dry matter intake (DMI), feed efficiency (FE) and fecal scores and antibiotic treatments. However, it seems these positive results might be indirectly or directly related to SCY improving the gut environment by decreasing interaction between pathogens and cells in the gastrointestinal tract of the calves [93] and hence the gut health [32]. Various SCY products have been linked to better rumen fermentation [35].

It remains a challenge for the researchers to conclude definitely what part of both ALY and YC contributes to the positive health benefits or by what mechanisms. Porosity in information on the components that affect the health of calves fed the products have contributed to the speculations. Both proponents of ALY and YC have often pointed out to the presence of isolated components such as β-glucans and mannan-oligosaccharides [94] contained in the yeast cell wall. The proponents of ALY argue that yeast cell competes with the pathogenic microbes for attachments sites hence inhibiting them from attaching to the gut wall. In poultry, it has been suggested that mannan-oligosaccharides agglutinate to the Type-1 fimbriae structures presented by the pathogenic bacteria hence inhibiting their colonization of the gut lumen [95]. These observations should be applied with caution in calves, since it has been shown that some strains of Enterohemorrhagic *Escherichia coli* utilize other mechanisms, specifically type III secreted proteins and cytotoxins and not type 1 fimbriae to colonize calves’ intestines and cause diarrhea [96]. On the other hand, YC proponents propose that its metabolites are utilized by the gut microbiota leading to reduced number of pathogenic microbiota. To support their case, in vitro research has shown that metabolites produced during the fermentation process of YC can inhibit pathogenic *E. coli* growth while stimulating non-pathogenic *E. coli* [49]. It is thought that in vivo the good commensal microbes would utilize the metabolites supplied by the SCFP and through competitive exclusion replace the pathogenic microbes.

Fewer researchers have focused on how SCY can stimulate an immune response in animals which can help unravel alternative modes of action. However, it is noteworthy that SCY has the potential to assist an animal that is experiencing physiological or environmental stress. The effect of supplementing SCY in calves on growth, performance and health have varied between adding in calf starter, milk or both. The variability in experimental designs might contribute to the differences in results reported. Geographical differences, farm specific pathogens, environmental factors, species and doses applied might all cause the differences in results reported. The rate of supplementation of the different products of SCY also remains to be determined which would further help determine improving the health of the calves.

## Conclusions

*Saccharomyces cerevisiae* yeast seems to offer benefits to calves through improved DMI, growth, feed efficiency and reduction in diarrhea in calves. Furthermore, subtle improvements are seen in gut related parameters. Specifically, SCY tended to enhance rumen fermentation (increased butyrate production) and rumen papillae growth. However, the merits seem obvious in animals that are under some form of stress. Several questions remain and further studies are required in order to gain a better understanding of the effects of SCY on calves. Areas that could be exploited in future include gut morphology, gut microbiology, and immunity using latest molecular methods like gene expression analysis. Furthermore, standardization of the research protocols needs to be taken into account. More experiments involving same products would result in a better database to carry out a meta-analysis which is considered better in analyzing effects of a product in animal models.

## Abbreviations

ALY: Active live yeast
DMI: Dry matter intake
ETEC: Enterotoxigenic *Escherichia coli*
FE: Feed efficiency
FPT: Failure of immune transfer
GIT: Gastrointestinal tract
IgG: Immunoglobulin G
NK: Natural killer
NLR: Neutrophil to lymphocytes ratio
PAMPs: Pathogen-associated molecular patterns
PRR: Pattern recognition receptors
ROS: Reactive oxygen species
SCFAs: Short chain fatty acids
SCFP: *Saccharomyces cerevisiae* fermentation product
SCY: Yeast of *Saccharomyces cerevisiae*
VCR: Crypt depth ratio
VFA: Fatty acids
YC: Yeast culture
